# To Subscribers

**Published:** 1867-03

**Authors:** 


### TO SUBSCRIBERS.

In consequence of the extreme illness, for the past ten weeks, of the senior editor of our Journal, who has in charge the financial department, there has been no recognition of scbscriptions received.

But, as we are happy to be able to state that he is rapidly recovering, receipts for moneys received for some months past will appear in the next number of the Journal. And in this connection, permit us to urge opon those who are in arrears to send at once, by letter or express, at our risk, their subscriptions for the last volume. It is a small amount to each individual subscriber, but the aggregate is extremely burthensome to us. Let each subscriber, then, make some sacrifice, if necessary, and send the amount due us; for we have made many, during the past year, in sending out the Journal.

